# Assessing the Power of Exome Chips

**DOI:** 10.1371/journal.pone.0139642

**Published:** 2015-10-05

**Authors:** Christian Magnus Page, Sergio E. Baranzini, Bjørn-Helge Mevik, Steffan Daniel Bos, Hanne F. Harbo, Bettina Kulle Andreassen

**Affiliations:** 1 Institute of Clinical Medicine, University of Oslo, 0316, Oslo, Norway; 2 Department of Neurology, Oslo University Hospital, 0424, Oslo, Norway; 3 Department of Neurology, University of California San Francisco, San Francisco, California, 94158, United States of America; 4 University Center for Information Technology, University of Oslo, 0316, Oslo, Norway; 5 Department of Research, Cancer Registry of Norway, 0304, Oslo, Norway; Children's National Medical Center, Washington, UNITED STATES

## Abstract

Genotyping chips for rare and low-frequent variants have recently gained popularity with the introduction of exome chips, but the utility of these chips remains unclear. These chips were designed using exome sequencing data from mainly American-European individuals, enriched for a narrow set of common diseases. In addition, it is well-known that the statistical power of detecting associations with rare and low-frequent variants is much lower compared to studies exclusively involving common variants. We developed a simulation program adaptable to any exome chip design to empirically evaluate the power of the exome chips. We implemented the main properties of the Illumina HumanExome BeadChip array. The simulated data sets were used to assess the power of exome chip based studies for varying effect sizes and causal variant scenarios. We applied two widely-used statistical approaches for rare and low-frequency variants, which collapse the variants into genetic regions or genes. Under optimal conditions, we found that a sample size between 20,000 to 30,000 individuals were needed in order to detect modest effect sizes (0.5% < PAR > 1%) with 80% power. For small effect sizes (PAR <0.5%), 60,000–100,000 individuals were needed in the presence of non-causal variants. In conclusion, we found that at least tens of thousands of individuals are necessary to detect modest effects under optimal conditions. In addition, when using rare variant chips on cohorts or diseases they were not originally designed for, the identification of associated variants or genes will be even more challenging.

## Introduction

Since the introduction of Genome Wide Association Studies (GWAS), a large number of common single nucleotide variants (SNVs) have successfully been associated to many complex diseases [1]. However, both the proportion of the phenotypic variability explained by these variants and the effect sizes are rather small for most studied traits. This issue has been widely discussed and is referred to as “missing heritability” [2–5]. This term suggests that genetic causes that are difficult to detect with a classic SNV array design are involved in the phenotype of interest. Such causes may be gene-gene and gene-environment interactions, chromosomal aberrations, epigenetic differences, or less frequent causal variants with minor allelic frequencies of 0.5% to 5% (low-frequency variants) or less than 0.5% (rare variants). Several of such rare and low frequent SNVs have been shown to associate with complex diseases with odds ratios (ORs) around 3 (e.g. [6–8]). Some structural variants associated to psychiatric disorders have been reported with even higher ORs (e.g. [9–12]). For example, a structural variant has been shown to give as much as a 20 fold increased risk for autism spectrum disorder [10].

The importance of considering allelic variants in coding regions, as well as budgetary and practical restrictions for whole exome sequencing in large studies, motivated the construction of the “exome chips” [13, 14]. A number of studies that used exome chips have already been published [15–29], with several of the studies reporting negative findings. However, phenotype-associations of some variants and genes have been discovered using this chip. Igartua *et*.*al*. [20] found one low-frequent variant associated to asthma when using a single variant test in a multi-ethnic cohort of 11,225 individuals. By using a collapsing approach (Sequence Kernel Association Test [30]), two additional genes were identified. Within a cohort of 8,229 Finnish individuals, Huyghe *et*.*al*. [16] identified new associations of low-frequent loci to fasting glucose levels. In a follow up case-control study by Wessel et.al., including more than 158,000 individuals, and by using statistical approaches designed for low-frequency or rare variants, one novel genetic association was discovered, driven by four rare non-synonymous SNVs within this gene [21]. With a multi-ethnic cohort of 56,000 individuals typed on the exome chip, four low-frequent variants were identified to be associated with coronary heart disease, using a single variant test. Furthermore, Tachmazidou *et*.*al*. identified a significant cardio-protective variant which was common in an isolated population, however this variant is assumed to be rare in outbred European populations [28].

The design of the exome chip was based on pooled exome sequencing data of 16 contributing studies [31], which comprised 12,031 individuals. These studies were highly enriched for European Americans, which accounted for approximately three-quarters of the sequenced individuals [20]. This has caused a concern concerning the generalizability of using low-frequency and rare variants in studies across populations. These variants are more likely to be evolutionary young [32], and thus, population specific. Approximately 65% of the contributing individuals were enriched for lifestyle disorders (Cardiovascular diseases, Type 2 Diabetes, Overweight, Lipid extremes, Body Mass Index extremes). Additionally, 20% of the samples were collected from psychiatric disorder cohorts (autism spectrum, schizophrenia and depression). The remaining 15% were samples from the thousand genomes project, a Sardinian cohort (SardiNIA sequencing project), and two cancer studies (S1 Table). In the design of this chip many common disease groups were absent, including autoimmune and neurodegenerative diseases. The exome chip consortia focused on capturing low-frequency and rare, non-synonymous variants, which were observed more than three times in at least two different cohorts. Most of the variants assayed on the exome chips were rare (84%), 9.2% were low-frequent, and 5.8% were common. Both the companies Illumina and Affymetrix produced a genotyping chip for low-frequent and rare, exonic variants based on the proposed list of SNVs from the Exome Chip Consortia, leading to the Illumina HumanExome BeadChip Array and the Axiom Exome Genotyping Array, respectively.

Since the power to detect an association between a single SNV and a phenotypic trait decreases with decreasing minor allelic frequency, there has been a need for new statistical tools for analysing low-frequent and rare variants. These variants often occur at different locations throughout the considered genes. Therefore, methods for this type of variants have been developed, which aim to collapse variants along a meaningful biological unit (i.e. gene, promoter, enhancer, etc.) into one test statistic. This includes methods which contrast the mean number of observed variants between cases and controls, such as Weighted Sum Statistic (WSS) [33] and Replication Based Test (RBT) [34], or adaptive burden tests, like the Kernel Based Adaptive Clustering Method [35]. Another general class of methods comprises variance contrasting methods, which compare the variation of alleles between cases and controls, such as the C(α)–method [36] and Sequence Kernel Association Test (SKAT) [30]. While several different methods have been compared extensively (e.g.[37–42]), no single gold standard has been established. On the contrary, it is also recommended to use different kinds of methods [37, 41].

With respect to the increasing use of exome genotyping chips, we aimed to investigate the sample size requirements for association studies using these chips. The power for different statistical approaches for analysing low-frequent and rare variants has been investigated and compared to each other by others [30, 33, 37, 40, 41]. The corresponding simulations were performed for varying properties of a single unit (i.e. gene), thereby focusing on the comparison of statistical methods with respect to the detection of rare and low-frequency variants. These simulations did not take the whole variety of possible allelic frequencies into account, neither the dependencies between the variants corresponding to a real chip design. Thus, these power simulations are only representative for certain allelic frequencies, ignoring the underlying realistic allele frequency distribution and dependency patterns.

We developed a simulation pipeline, which relies on simulations based on all variants of the underlying chip design, thereby capturing the entire allele frequency spectrum and underlying dependencies between the variants. In this paper, we mimicked the structure of the Illumina HumanExome BeadChip array, but the available pipeline can also be applied to any other (future) chip designs.

## Material and Methods

### Simulation of genotypes

As a starting point for the simulations in this paper, we simulated a data pool of genotypes for 200,000 unrelated individuals using the approach described in Basu *et*.*al*.[41], with some modifications. To mimic the chip as accurately as possible, we used the publicly available allele frequencies reported by the Exome Chip Consortia. From their documentation [31], we reproduced the allele frequency of 212,353 non-synonymous SNVs, thus including 96% of the coding variants on the chip. In order to mimic the dependency structure between the variants, we applied a correlation function based on the position of the variants on the exome chip [43].

### Simulation of phenotypes

To construct case-control phenotypes, we used the same approach as Madsen *et*.*al*. [33, 37] fixating the population attributable risk (PAR) for all variants, and calculating a genotype relative risk (GRR) based on the given PAR and the minor allele frequency (MAF) (see S1 Algorithm Eq 1). We assumed that all causal rare variants were deleterious, and that no variants had any protective effect. The probability of an individual being diseased based on their genotype, was calculated as the product of their GRRs, multiplied by a fixed incidence (see S1 Algorithm Eq 2). This was done for each individual separately. The relation between GRR and PAR is such that for a given MAF, and PAR, a linear increase in PAR corresponds to a linear increase in GRR. If the PAR was fixed, then an increase in the MAF corresponded to an inverse proportional decrease in GRR.

We considered two different scenarios for the structure of the simulated causal genes. In the first scenario, 100% of the SNVs in each analysed gene were causally linked to the phenotype. In the second scenario, the same genes were analysed, but only 50% of the SNVs within each gene where causally linked to the phenotype, thus decreasing the signal to noise ratio.

### Statistical Methods

To assess the sample size required to obtain sufficient power, we applied two widely used statistical methods for rare variants: SKAT [30] and WSS [33]. In SKAT which is a generalization of the variance contrast test (C(α) method [36]), we used an adaptive weighting for each variant (the *Beta*(MAF, 0.5, 0.5) kernel). The WSS test is an adaptive sum test, for each unit, it calculates a weighted sum for all individuals, and then permutes the ranking of those sums, if the cases are consistently ranked on top, this will correspond to a low p-value. The weight for each variant is determined by the MAF and the case-control ratio. The two statistical methods used here were chosen as representatives for two common classes of methods for rare variant analysis; variance contrasting tests and sum tests. In both methods, all the genes are tested independent of each other. The weights applied to all variants have similar structure for both SKAT and WSS. In both methods, the weighing is such that common alleles will receive a low weight, while empirically rare variants will have a high weight.

#### Power Simulations

We investigated the power performance by drawing sample sizes of 10,000 (10k), 20k, 30k, 60k, and 100k individuals from the genotype pool described above. The simulated case-control ratio was 1:1. To assess the power under the different scenarios, we randomly selected a set of 100 genes. The distribution of allelic frequencies of this subset was similar to the corresponding allelic frequency distribution of all SNVs on the chip (S1A Fig). The mean number of SNVs per drawn gene was 18. 50 simulated datasets including 100 genes were generated for each combination of effect size, scenario and number of individuals. For each simulated dataset, the genes were tested on the Bonferroni adjusted genome-wide threshold based on the number of reproduced genes on the chip (19,975), thus neglecting findings in the genes without any simulated effect. The power was defined as the percentage of true discovered genes within one replicate. The overall power was presented as the mean power over all replications along with the empirical 95%—confidence interval.

#### Null simulations

We provide two types of simulations without adding an effect on the simulated genotypes (0% PAR on all causal variants). First, we aim to characterize the implemented statistical methods with respect to their ability to detect false positive findings. To achieve that, we used the 50 simulated datasets for 60k samples including 100 genes described above and assigned case-control status randomly. For each simulated dataset, we evaluate the percentage of false positives and present the mean percentage across all simulated datasets. In this simulation, we choose a 5% threshold for the p-values of each gene. A genome-wide threshold could have been simulated here as well, but would require a much larger number of null genes and thus dramatically increase the computational burden. Second, we wanted to show the genome-wide performance of the tests with no underlying effect present for the underlying chip structure considered in this paper. Thus, we simulated 10 datasets including all genes (19,975) for two different numbers of individuals (10k, 60k), assigning the case-control status arbitrarily.

The simulations and power assessment where done using the computer program R 3.2.1 [44], with the additional packages: Matrix [45], MultiPhen [46] and snpStats [47].

The simulation program can be received from the authors by request.

## Results

**Fig 1:**
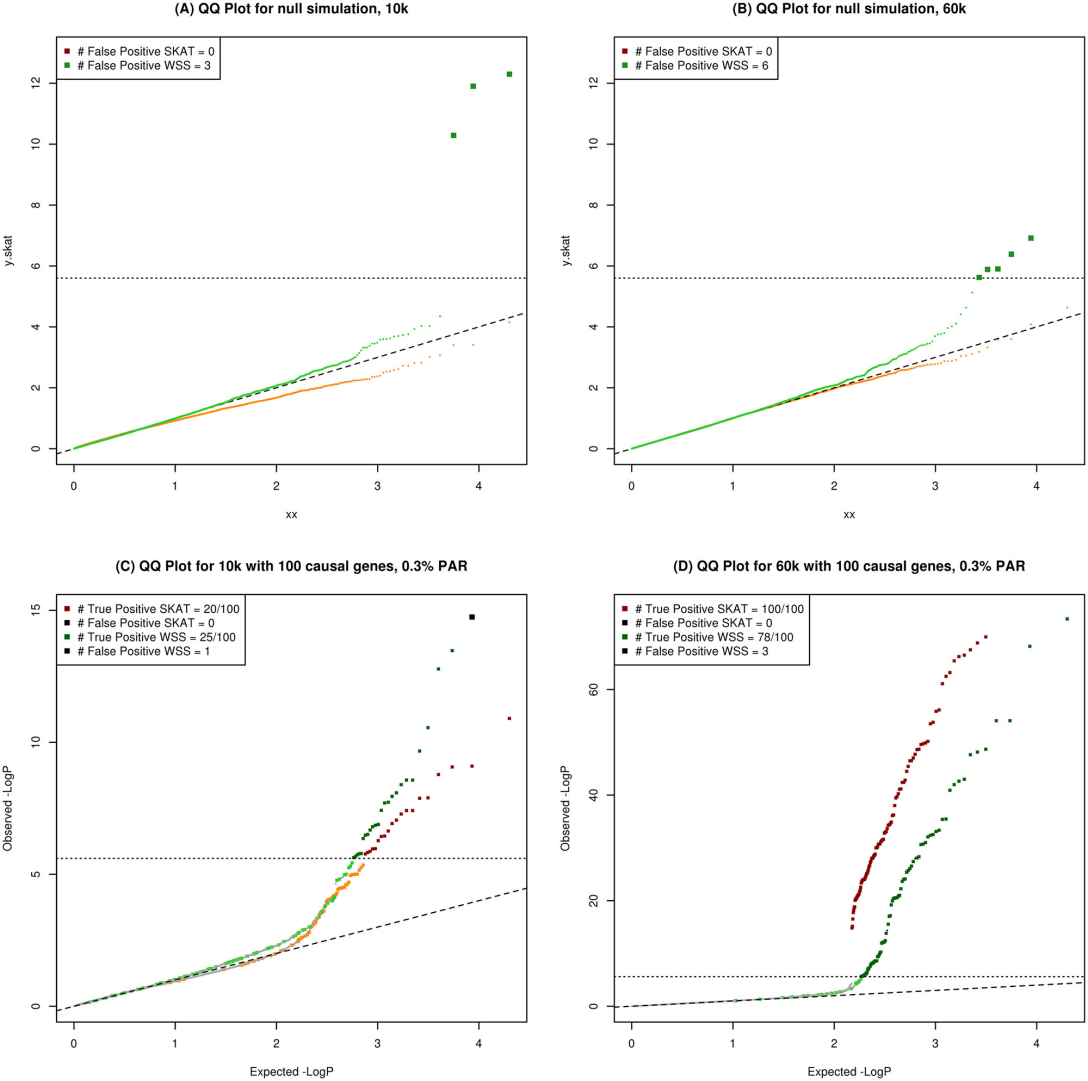
QQ-plot for power analysis and null simulation, the diagonal line represent the expected value and the horizontal line the Bonferroni cut-off. (A) QQ-plot for one realization of the null simulation for 10k, SKAT is plotted in red/orange and WSS in dark green/light green. (B) QQ-plot for one realization of the null simulation for 60k, SKAT is plotted in red/orange, and WSS in dark green/light green. (C) QQ-plot of—log p-values for SKAT and WSS, given 100% causal SNVs within the causal genes, and a sample size of 10k. False negative is in lighter colors (SKAT; light green, WSS; orange) and true negative is colored in gray. (D) QQ-plot of—log p-values for SKAT and WSS, given 100% causal SNVs within the causal genes, and a sample size of 60k. False negative is in lighter colors (WSS; orange) and true negative is colored in gray.

Both of the statistical methods keep the Type-I error level, with SKAT being slightly more conservative than WSS. The corresponding estimated mean percentages of false positives were 0.0465 (SKAT) and 0.0503 (WSS) when applying the 5% Type-I error threshold (see Material and Methods). In order to understand the performance of the exome chip when no effects are present, the distribution of the p-values across all 19,975 genes was visualized in a QQ-plot (Fig 1A and 1B). It can be seen that SKAT is more conservative, with no false positive observations, while WSS had an average of 4.7 false positive for a 10k sample, and an average of 5.4 false positive in a 60k sample, in a genome wide scan.

**Fig 2:**
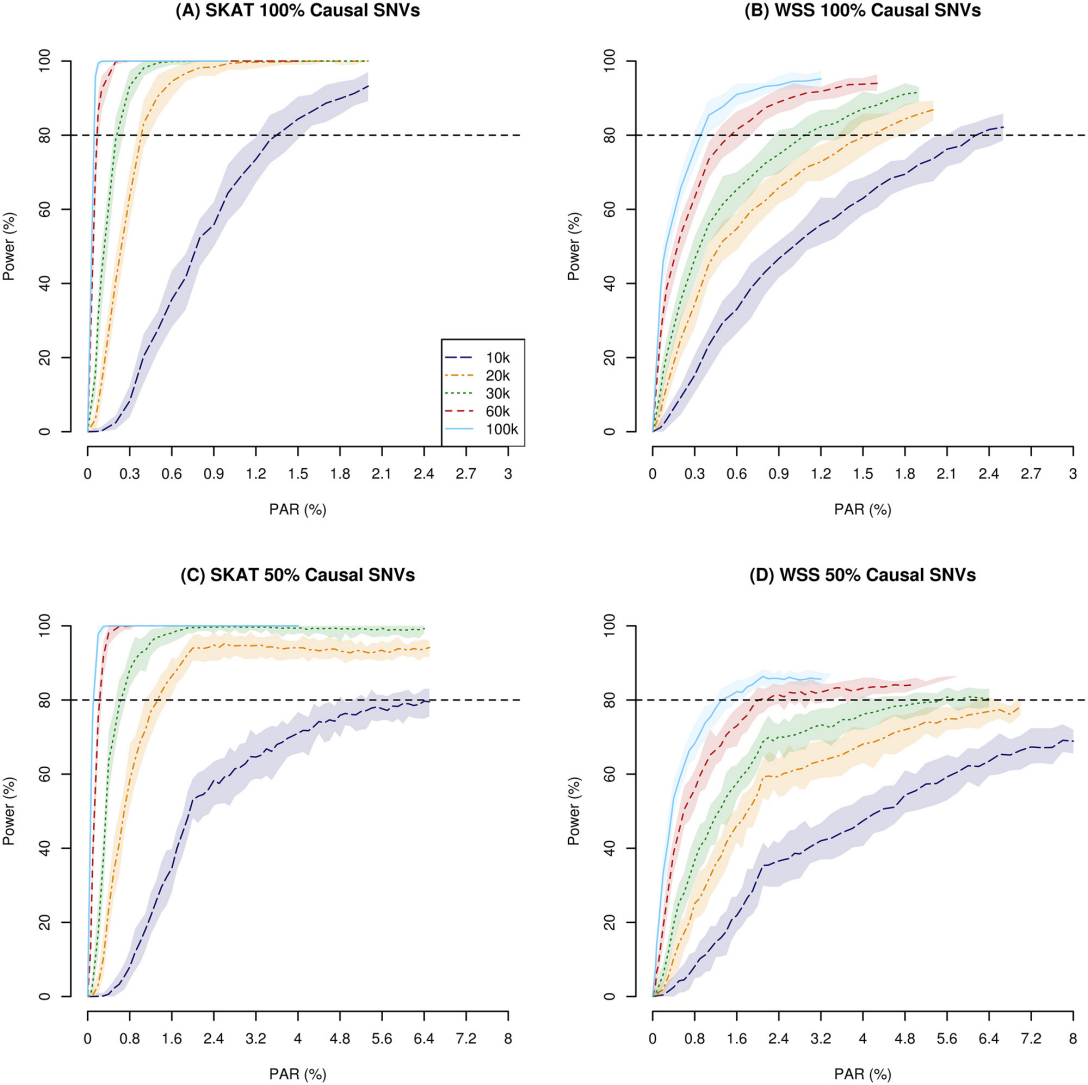
Power plots for increasing PAR for SKAT and WSS for multiple testing adjusted analyses, for different sample sizes. The dashed line represent the median power, with the covered area is the inter quantile range of 25% to 75% power. (A) 100% causal SNVs within all genes, estimated with SKAT. (B) 100% causal SNVs within each gene, estimated with WSS. (C) 50% causal SNVs within each gene, estimated with SKAT. (D) 50% causal SNVs within each gene, estimated with WSS.

To assess the power of the underlying chip under the non-null distribution, we simulated an increasing effect size (PAR) for different sample sizes based on two statistical approaches (SKAT and WSS). We first investigated a scenario where all variants within a gene were causal. In this scenario, both SKAT and WSS reached a power of 80% with a PAR less than 1.4% (SKAT) and 2.4% (WSS) per SNV, and SKAT converged to maximum power for 1.5% for sample sizes above 10k (Fig 2A and 2B). SKAT and WSS had approximately the same speed of convergence when all variants were assigned the same weight in SKAT (data not shown). For sample sizes larger than 20k individuals, the rate of convergence of power evolved more than twice as fast in SKAT as compared to WSS. The increase in power for sample sizes above 60k individuals was marginal in SKAT. However, in WSS, the rate of convergence between the different sample sizes was more pronounced, with notable differences in convergence for sample sizes of 60k and 100k individuals. For small effect sizes (PAR < 0.5%) and a sample size of 10k, WSS converged marginally faster than SKAT. To evaluate the global performance of the chip for a given PAR in this scenario, we applied both WSS and SKAT to all genes, with a sample size of 10k and 60k. The result for PAR = 0.3% on all causal variants is presented in Fig 1C and 1D. Fig 1 shows that SKAT is more conservative in its p-value estimation than WSS, both for the null simulation and with an effect size of 0.3% PAR for a sample size of 10k.

When only 50% of the SNVs within each unit were causal, a much slower convergence was observed for both methods (Fig 2C and 2D). For 10k individuals, a PAR of 6.5% was needed to obtain 80% power with SKAT, whereas WSS reached 70% power within 8.0% PAR on each causal variant. To reach 80% power with WSS within 6% PAR, a sample size of at least 30k was needed. For SKAT, a sample size of at least 30k did converge to 100% power for PAR up to 7%. This is a substantial loss of power, compared to the assumption that all SNVs within each gene were causal. In that case, half of the effect sizes were sufficient to reach the same power. When considering sample sizes larger than 10k individuals, 80% power is reached within a PAR of 1.4% for SKAT. WSS reached 80% power within 5.6% PAR for sample size of 30k. For sample sizes above 10k, SKAT converged to maximum power at 2% PAR. In WSS, the convergence was substantially slower, with none of the sample sizes converging to 100% within their tested range of PAR.

**Fig 3:**
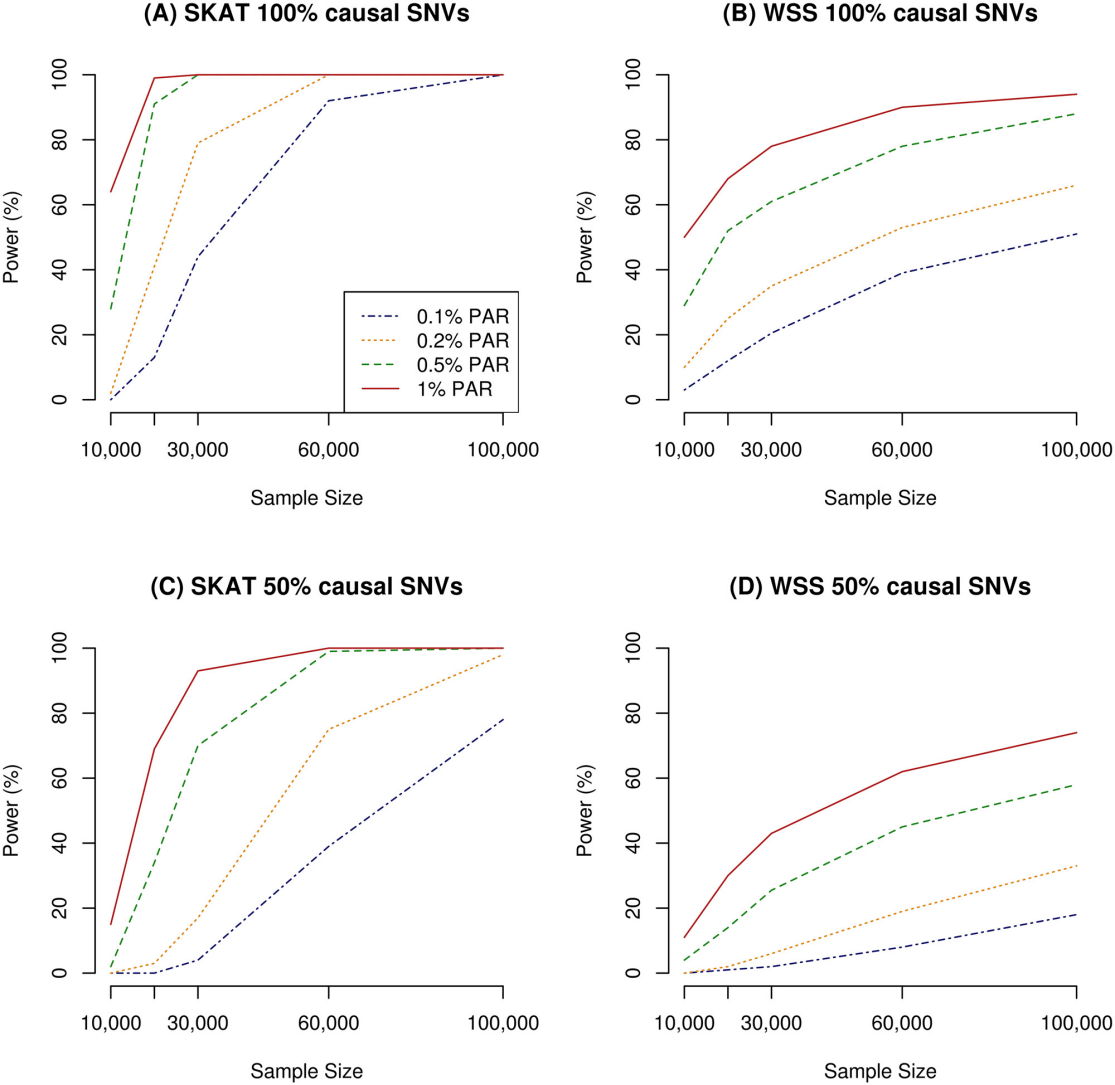
Power for increasing sample sizes and different PAR values after multiple testing adjusted analyses. (A) 100% causal SNVs within all genes, estimated with SKAT. (B) 100% causal SNVs within each gene, estimated with WSS. (C) 50% causal SNVs within each gene, estimated with SKAT. (D) 50% causal SNVs within each gene, estimated with WSS.

In order to assess how many individuals would be needed for a given power, we plotted power as a function of sample size (Fig 3). Under the assumption of 100% causal variants per unit, the best performance was reached with a sample size of 60k individuals or more, where both SKAT and WSS were above the 80% threshold in for the two biggest effect sizes presented (PAR = 0.5% and 1%). For WSS, a larger sample size was consistently needed to obtain the same power as SKAT in the same scenarios (Fig 3A and 3B). When 100% of the SNVs were causal, the power of WSS was comparable to the power of SKAT when only 50% of the variants were causal (Fig 3B and 3C). For effect sizes of 0.2% PAR in the 50% scenario, a sample size of 60k was sufficient to reach 80% with SKAT, but for WSS, 100k was needed (Fig 3C and 3D).

In order to investigate the relationship and distribution of the effect sizes GRR and PAR of the causal variants, we plotted a histogram of the GRR for different PAR (S1B Fig). For a fixed PAR of 0.5% on all causal variants, the GRR range in our simulated data was between 1 and 70, with a median of approximately 12. Since the GRR scales linearly for small PAR, a doubling of PAR to 1%, resulted in a doubling of the GRR. The corresponding GRR then had a maximum of 140 and a median of 24, as seen in S1B Fig.

## Discussion

In this work we addressed the utility of genotyping chips for rare variants under optimal conditions, illustrated by simulating the content of the Illumina HumanExome BeadChip array under different scenarios. Given a homogenous population (as was used for the design of the chip), we found that to detect a true association with 80% power, for a PAR around 1.5% on each causal variant in the presence of noise, a sample size of at least 20k individuals were needed under optimal conditions. Thus, the chip performance was acceptable for large (PAR > 1%) effects even in relatively small cohorts (10-20k). For small effect sizes (PAR < 0.5%) in the presence of noise, a balanced case-control study with a total sample size of 30k to 50k individuals would be required.

Our assumption of sample homogeneity of conferred risk for the SNVs in this analysis is not likely to be met in most association studies. This is mainly due to population specific rare variants. We also assumed that all rare coding causal variants were deleterious. Although some variants may be protective, the majority of rare coding alterations are believed to be either harmful or have low phenotypic effect [14], thus making our assumption a reasonable choice. We have focused our simulations on two different scenarios, one where 100% of the assayed alleles within the gene were deleterious, and the other where 50% of the alleles were deleterious. A scenario where all detected variants within a gene are causal to disease is very unlikely, but represents an upper bound on the power estimate for collapsing methods. In earlier studies which identified associated genes, the fraction of causal rare and low-frequent SNVs within the gene was estimated to be as low as 5% [29]. For the gene discovered to be associated with psychophysiological endophenotypes by Vrieze *et*.*al*. the association seemed to be driven by two alleles, which represent 10% of the variants in this specific gene on the exome chip [27]. One of these variants was low-frequent (MAF = 1.25%) and the other one rare (MAF = 0.3%) [27]. 40% of the rare coding exome chip variants within a gene associated to higher fasting glucose levels showed a strong individual association to this phenotype [21].

An important issue is the enrichment of SNVs associated with certain diseases in the design of the exome chip. Many common complex disease groups were not represented in the cohorts used to design the chip, leaving the possibility that rare variants which may be strong risk factors for these diseases were not included on the chip. The rest of the SNVs included may be neutral or have very small phenotypic effects. This will most likely result in sub-optimal performance in studies of diseases that were not considered when designing the exome chips.

Although the difference in mean power between SKAT and WSS analyses was not substantial, SKAT consistently outperformed WSS, which is in line with previous studies [37]. Furthermore, SKAT also outperformed WSS on elapsed computational time, where the R implementation of SKAT could benefit from parallelization on a cluster computer infrastructure.

The simulation pipeline developed here could be adapted to different chip designs. This program is only dependent on allele frequencies and positions, since the linkage disequilibrium (LD) between variants was modelled with a distance function. Furthermore, the algorithm is flexible in its implementation, so it can be applied to assess the performance of any other chip design, under different scenarios. In some simulation studies for assessment of rare variant methods, a genome wide p-value cut-off was not used [37, 41] and the allele frequency range was much wider. Simulations were performed on a single unit (i.e. genes) which included several variants, leaving out valuable information about realistic underlying allele frequencies and dependency patterns. In our study, we mimicked the properties of the exome chip, increasing both usability and reliability of our results.

There is no standard algorithm for simulating effects on genetic variants, this has led to a situation where the reported results can vary depending on the implemented methods and assumptions. Two popular approaches for emulating effect sizes are Odds Ratio (OR) modelling and Risk Ratio (RR) modelling. Although these approaches are quite different, when the number of observed genotypes is small, both the OR and the RR will be approximately the same.

When the GRR was empirically estimated from the simulated data set, they were consistently lower than expected from the equation used to generate them (S1 Algorithm Eq 1). This indicates that the GRR presented in S1B Fig was overestimated, since it is theoretically calculated, and not empirically assessed.

The collapsing methods tests each gene (unit) and the power presented in Figs 2 and 3 on the y-axis are gene-wise. However, the effect applied on the genotypes (x-axis in Fig 2), was per SNV and not per gene. When considering genes, it is important to note that the disruption of any coding element may be contributing to disease risk, and different variants within a gene can all disrupt the gene product, with observed mild effect sizes for each variant. For this reason, many different variants within the same gene may be underlying the same trait or disease. By using the collapsing statistic on a gene instead of testing individual variants, the sample sizes requirement may therefore be smaller. By selecting genes at random in the data simulation process, we study the variety of genes on the chip, without using the entire data set, thereby decreasing the computational load. Since the underlying allelic frequencies were properly presented, this gives a good indication of the overall performance of the chip, however, the actual performance for each particular gene may vary from gene to gene.

While recent studies using exome chips have identified associations between low-frequent or rare variants and disease, the identified variants have not yet contributed substantially to explaining “The missing heritability”. Few of the studies have reported variants with minor allele frequency below 0.5% to be associated with disease [21, 24, 26–28]. Our study indicates that some negative reports may suffer from insufficient sample sizes and the special design of the exome chips as explained above.

In our study we have only considered “perfectly called” variants, i.e. we have not introduced any errors in the genotype calling algorithms. This may be an important issue for rare and low-frequency genotyping chips, where calling the variants has proved to be challenging [48].

In conclusion, we found that a very large sample size, in the order of tens of thousands is needed to detect modest effects under optimal conditions. For effect sizes less than 0.2% PAR, around 100,000 individuals should be studied to have enough power to reach genome wide significant results.

## Supporting Information

S1 Table Reproducing the table from the exome chip consortia [31], showing the different contributions to the design of the exome Chips. (DOCX)

S1 Fig (A) Histogram of the distribution of the allele frequencies on the exome chip, plotted on log 10 scale. The histogram is split into three bins, depending on their allele frequency. The lines indicated the allele frequencies of the causal alleles in each scenario. The blue line represents the allele distribution of the SNVs selected in the scenario where 100 of the SNVs within each causal gene were causal. The orange line represents the allele distribution of the causal SNVs, where 50% of the SNVs within the casual genes are causal. (B) Histogram of Genotype Relative Risk (GRR) for all causal variants in the 100% scenario, for two different Population Attributable Risks (PAR). This is the GRR used to construct the phenotypes for those two PARs.
(TIF)

S1 Algorithm Algorithm for variant simulation and phenotype construction. (DOCX)

S1 Source Code R code for reproducing the variant simulation and phenotype construction. (TAR.GZ)
