# Supplementary material for: Assessing the Power of Exome Chips
Source: PLoS One. 2015 Oct 5;10(10):e0139642. doi: 10.1371/journal.pone.0139642 (PMC4593624; doi:10.1371/journal.pone.0139642)
Supplement: S1 Table — (DOCX) [file pone.0139642.s001.docx]

**Supplementary table 1** reproducing the table from the exome chip consortia (Exome Chip Consortia), showing the different contributions to the design of the exome Chips.

| Study name | Phenotype | Decent | Sample Size |
| --- | --- | --- | --- |
| NHLBI ESP (5 tranches) | CD, Lung traits, Obesity | (American) Europeans, AA | 4260 |
| ARRA | Autism | (American) Europeans | 1778 |
| GO T2D (2 tranches) | T2D | (American) Europeans | 1618 |
| KG (2 tranches) | Healthy individuals | Diverse | 1128 |
| Sweden Schizophrenia Study | Schizophrenia | European (Swedes?) | 525 |
| SardiNIA | LDL-c, HDL-c, TG | Sardinia population | 508 |
| CoLaus | Overweigh, Diabetes, Fasting Glucose | European (UK) | 456 |
| Cancer Genome Atlas | Cancer | European | 422 |
| T2D GENES | T2D | Hispanic (Mexico) | 362 |
| Cancer Cohort Study (SMWHS*) | Cancer | Chinese | 327 |
| Pfizer/MGH/Broad | T2D Extreme risk | European | 182 |
| Lipid Extremes | Lipid Extremes | European | 131 |
| Int’l HIV Controllers | HIV Controllers | (American) Europeans | 121 |
| SAEC DILI | Augmentin DILI | European | 117 |
| I2B2 | Major Depression | European | 50 |
| BMI Extremes | BMI Extremes | European | 46 |

*SMWHS= Shanghai Men and Women Health Study
