# Supplementary figures and images for: Assessing the Power of Exome Chips
Source: PLoS One. 2015 Oct 5;10(10):e0139642. doi: 10.1371/journal.pone.0139642 (PMC4593624; doi:10.1371/journal.pone.0139642)

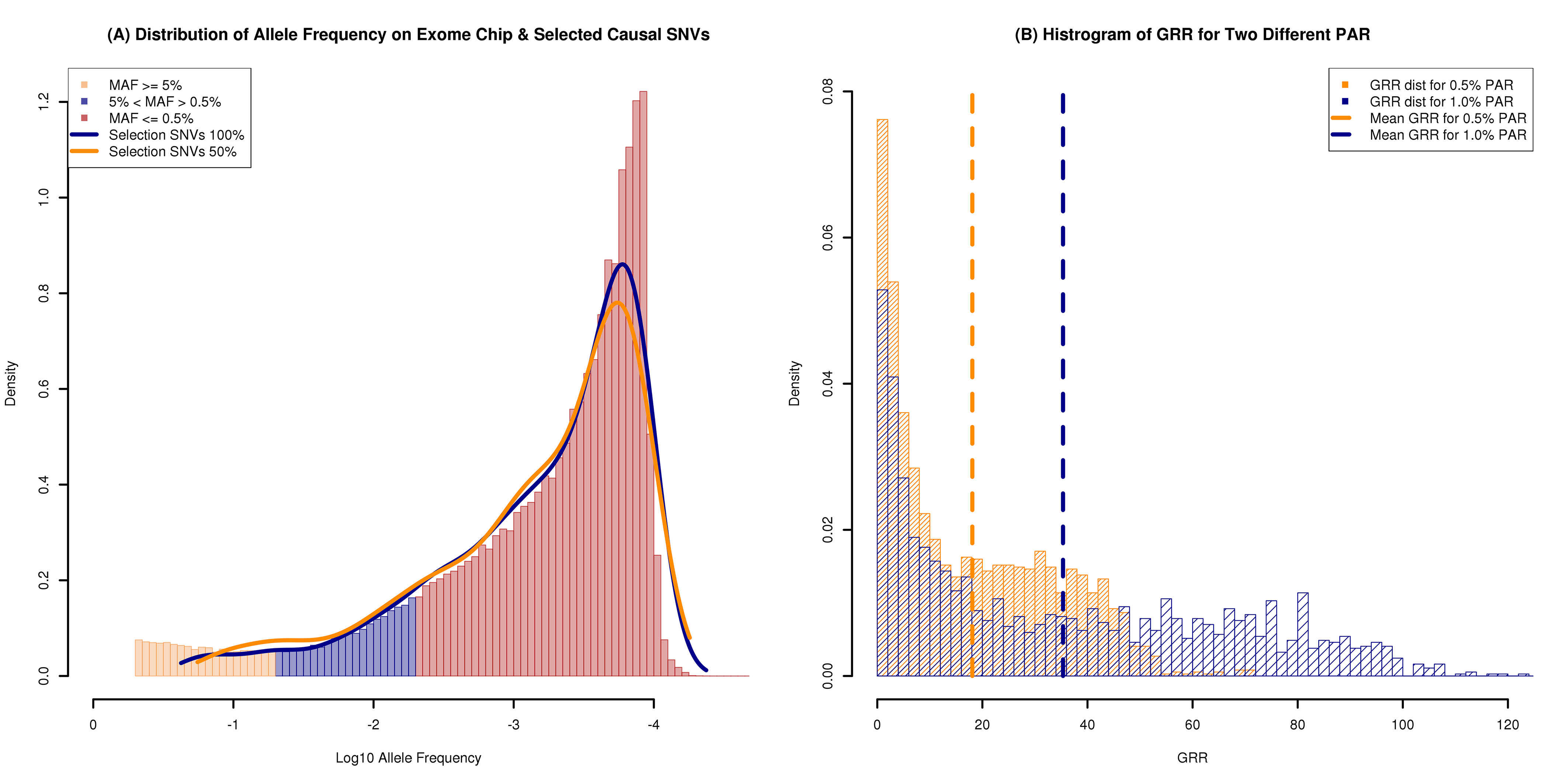

Supplement: S1 Fig — (A) Histogram of the distribution of the allele frequencies on the exome chip, plotted on log 10 scale. The histogram is split into three bins, depending on their allele frequency. The lines indicated the allele frequencies of the causal alleles in each scenario. The blue line represents the allele distribution of the SNVs selected in the scenario where 100 of the SNVs within each causal gene were causal. The orange line represents the allele distribution of the causal SNVs, where 50% of the SNVs within the casual genes are causal. (B) Histogram of Genotype Relative Risk (GRR) for all causal variants in the 100% scenario, for two different Population Attributable Risks (PAR). This is the GRR used to construct the phenotypes for those two PARs. (TIF) [file pone.0139642.s002.tif]
