# Supplementary material for: Assessing the Power of Exome Chips
Source: PLoS One. 2015 Oct 5;10(10):e0139642. doi: 10.1371/journal.pone.0139642 (PMC4593624; doi:10.1371/journal.pone.0139642)
Supplement: S1 Algorithm — (DOCX) [file pone.0139642.s003.docx]

### Algorithm for Simulation and Phenotype Construction:

1. We constructed a set of 2*N=400,000 independent multivariate normally distributed vectors, of length p=212,353 (representing each SNV). The correlation within the vectors, where modeled using the Matern covariance function, with parameters (sigma, phi, rho) equal to (1.9, 10, 15) respectively.
2. All the 2*N vectors was dichotomised with a threshold, using the allele frequency for each SNV, reported by the exome chip consortia. The vector now represents the independent maternal and paternal haplotypes.
3. The vectors were added together in groups of two, resulting in data set of N vectors, representing each individual, with the number of alleles (Basu and Pan 2011).
4. The alleles were now clustered together into their respective genes.
5. We selected a set of 100 genes at random to be causally linked to the phenotype
6. Two scenarios were constructed. In the first scenario, all SNVs within the select causal genes, where themselves causal. In the second scenario, we picked 50% of the SNVs within the causal genes at random to be causally linked to the phenotype.
7. For a given Population Attributed Risks (PAR), we calculated the Genotype Relative Risk (GRR) for each causal variant, using the equation below. Observe that this equation is only dependent on the PAR and the allele frequency, which is considered the exposure of the allele. (Equation 1)

$${GRR}_{j}=\frac{PAR}{\left( 1-PAR \right){MAF}_{j}}+1$$

8. Given the set of *k* GRRs for one PAR, we calculated the probability for each individuals of being a case, using the equation below. This resulted in a vector of N entries with either zero or one, corresponding to the phenotype for each individual. (Equation 2)

$$P\left( affected|Genotype \right)=b_{0}\prod_{j=1}^{k} {GRR}_{j}^{{AC}_{j}}$$

Here b_0_ is the base line population risk (incidence), *k* is the number of causal alleles, and AC_j is the allelic count of the allele number *j*, {0,1,2}.
9. For a set of increasing PAR (p1,p2,…,p_m ), we calculated the corresponding phenotype vector, giving a matrix of dimension N*m.
10. The genotypes for the causal genes along with the set of different phenotypes, where given to SKAT and WSS
11. For each phenotype vector (corresponding to all the PAR analysed), drew a random subset of a given sample size and asses the detection percent over all genes, and repeated this for 50 replicates. We then calculated the mean and 95% empirical confidence interval of the power over all the replications.

**Relationship between PAR and GRR**

The relationship between Population Relative Risk (PAR) and Genotype Relative Risk (GRR) used in the simulation is clarified in the equations below. If we define PAR as a ratio between an Exposure (E) and the Relative Risk (RR), as in the formula below:

$$PAR= \frac{E \left( RR-1 \right)}{1+E \left( RR-1 \right)}$$

We can now substitute the Relative Risk with the Genotype Relative Risk (GRR), and the Exposure is substituted with the minor allele frequency (MAF), after some rearrangement we get

$$PAR= \frac{MAF \left( GRR-1 \right)}{1+MAF \left( GRR-1 \right)}$$

$$PAR \left( 1+MAF \left( GRR-1 \right) \right)=MAF \left( GRR-1 \right)$$

$$GRR \left( MAF*PAR-MAF \right)=MAF\left( PAR-1 \right)-PAR$$

$$GRR=1- \frac{PAR}{MAF \left( PAR-1 \right)}$$

This leads to the final relation which is used in the simulation alforithm;

$$GRR= \frac{PAR}{MAF \left( 1-PAR \right)}+1$$

The GRR can also be inverted to emulate a protective effect, by substituting the effect sizes with 1/effect size

$$GRR= \left( \frac{PAR}{MAF \left( 1-PAR \right)}+1 \right)^{-1},$$

## Phenotype Construction

To construct phenotypes, we calculated the probability of an individual being affected as the product of all the GRRs, given in the equation below(Equation 2)

$$P\left( affected | Genotype=\left\{ a \right\} \right)=\min\left\{ 1,b_{0}\prod_{j=1}^{k} {GRR}^{a_{j}} \right\}$$

Where b0 is the baseline risk (incidence), and *a* is the set of all causal alleles, such that aj is the allelic count (0, 1, 2), for allele number j.

To justify Equation 2 that the probability of being affected is the multiplication of GRR, we start with the (Genotype) Relative Risk for one variant, given an exposure (E), which can also be an allele:

$$RR= \frac{P\left( Y=1 | E \right)}{P\left( Y=1 | E^{c} \right)}$$

$$P\left( Y=1 | E \right)=RR\times P(Y=1|E^{c})$$

Now the last part of the equation (P(Y|E^c^)) is the probability of being affected given no exposure, which is the incidence or background risk (b_0_). For many different variants, we can assume that the probability of being affected is the intersection of the probabilities. If all the variants act independently, and the incidence is assumed to be constant for the trait, this reduces to multiplication of all relative risks:

$$\bigcap P\left( y=1 | E_{i} \right)=\bigcap_{\forall i} {RR}_{i}\times P\left( Y | E_{i}^{c} \right)=b_{0}\prod_{\forall i} {RR}_{i}$$
